# Detrimental Effects of Doping Al and Ba on the Thermoelectric Performance of GeTe

**DOI:** 10.3390/ma11112237

**Published:** 2018-11-11

**Authors:** Bhuvanesh Srinivasan, Alain Gellé, Jean-François Halet, Catherine Boussard-Pledel, Bruno Bureau

**Affiliations:** Univ. Rennes, ISCR UMR 6226, IPR UMR 6251, CNRS, 35000 Rennes, France; alain.gelle@univ-rennes1.fr (A.G.); jean-francois.halet@univ-rennes1.fr (J.-F.H.); catherine.boussard@univ-rennes1.fr (C.B.-P.); bruno.bureau@univ-rennes1.fr (B.B.)

**Keywords:** Thermoelectrics, GeTe, Al-doping, Ba-doping, loss of band convergence, lowered *zT*

## Abstract

GeTe-based materials are emerging as viable alternatives to toxic PbTe-based thermoelectric materials. In order to evaluate the suitability of Al as dopant in thermoelectric GeTe, a systematic study of thermoelectric properties of Ge_1−x_Al_x_Te (*x* = 0–0.08) alloys processed by Spark Plasma Sintering are presented here. Being isoelectronic to Ge_1−x_In_x_Te and Ge_1−x_Ga_x_Te, which were reported with improved thermoelectric performances in the past, the Ge_1−x_Al_x_Te system is particularly focused (studied both experimentally and theoretically). Our results indicate that doping of Al to GeTe causes multiple effects: (i) increase in *p*-type charge carrier concentration; (ii) decrease in carrier mobility; (iii) reduction in thermopower and power factor; and (iv) suppression of thermal conductivity only at room temperature and not much significant change at higher temperature. First principles calculations reveal that Al-doping increases the energy separation between the two valence bands (loss of band convergence) in GeTe. These factors contribute for Ge_1−x_Al_x_Te to exhibit a reduced thermoelectric figure of merit, unlike its In and Ga congeners. Additionally, divalent Ba-doping [Ge_1−x_Ba_x_Te (*x* = 0–0.06)] is also studied.

## 1. Introduction

The generation, storage and transport of energy are among the greatest challenges, if not the most formidable challenge of all, for years to come. In this regard, thermoelectric (TE) materials and devices have drawn increasing interest and attention due to their potential to reversibly convert waste heat into electricity [1]. The TE material’s efficiency is quantified by a dimensionless figure of merit, *zT* = *S*^2^*σT*/*κ* where *S*, *σ*, *T* and *κ* are the Seebeck coefficient, electrical conductivity, absolute temperature and total thermal conductivity (sum of the electronic part, *κ*_e_, and the lattice part, *κ*_latt_), respectively. Seebeck coefficient, electrical and thermal conductivity are inter-locked and there is a high degree of challenge to decouple the electronic and thermal transport [2]. To tackle these challenges, thermoelectric material research has recently flourished with the emergence of novel concepts of band engineering, nanostructuring and discoveries of various novel materials. Amongst the state-of-the-art TE materials, the extensively studied PbTe-based materials are limited by their toxicity for any practical applications, despite their high *zT* [3,4,5,6]. Recently, GeTe-based materials have emerged as a clear alternative choice, as they have proven to exhibit higher performance (*zT* > 1), if optimally doped with suitable elements [7,8,9,10]. Some of the strategies for GeTe-based materials to enhance the power factor (*S*^2^*σ*) and/or to suppress *κ*_latt_ were adopted on compositions such as GeTe-AgSbTe_2_ (TAGS) [11], GeTe-LiSbTe_2_ [12], GeTe-AgInTe_2_ [13],GeTe-AgSbSe_2_ [14], (GeTe)_n_Sb_2_Te_3_ [15], Ge_1−x_Pb_x_Te [16], Ge_1−x_Bi_x_Te [17], (Bi_2_Te_3_)_n_Ge_1−x_Pb_x_Te [18], Ge_1−x_In_x_Te [19], GeTe_1−x_Se [20], Ge_1−x_Sb_x_Te [21], Ge_1−x_Ag_x_Te [7], Ge_1−x_Mn_x_Te [22], Ge_1−x−y_Sn_x_Pb_y_Te [23], Ge_1−x_Sb_x_Te_1−y_Se_y_ [24], GeTe-GeSe-GeS [25], Ge_1−x−y_Bi_x_Sb_y_Te [26], Ge_1−x−y_Bi_x_In_y_Te [9], Ge_0.9-y_Pb_0.1_Bi_y_Te [27], and more recently Ge_1−x−y_Ga_x_Sb_y_Te [8]. The crystal structure of these GeTe-based compounds undergoes a second-order ferroelectric structural transition from rhombohedral symmetry (low temperature phase) to cubic symmetry (high temperature phase) at around 700 K [10].

This work tries to explore the suitability of trivalent Al and divalent Ba as dopants for improving the thermoelectric performance of GeTe. The choice of Al is particularly interesting, as its isoelectronic group-13 counterparts In and Ga, if doped in optimum concentration, have proven to strongly enhance the thermoelectric performance of GeTe [8,19].

## 2. Materials and Methods

The samples Ge_1−x_Al_x_Te (*x* = 0–0.08) and Ge_1−x_Ba_x_Te (*x* = 0–0.06) were synthesized by vacuum sealed-tube melt processing. The obtained ingots were crushed into powder and consolidated by Spark Plasma sintering, SPS (FCT Systeme GmbH) at 773 K for 5 min under an axial pressure of 60 MPa. Details pertaining to experimental procedures, and materials characterization including electrical and thermal transport property measurements were discussed in detail in our previous works [6,7,8,9,28,29,30].

Density Functional Theory (DFT) calculations were performed to understand the effect of doping on the electronic states of GeTe. We used the projector-augmented-wave (PAW) approach [31] implemented in the Vienna ab initio simulation package (VASP) [32]. Calculations were performed using the generalized gradient approximation (GGA) for the exchange-correlation term parametrized by J. P. Perdew et al. [33] Similar to our previous work on Ga-doped GeTe [8], spin orbit coupling was included in the computations. As we were interested in high temperature behavior of doped GeTe, calculations were performed on cubic structural models. Impurities were substituted to Ge atom in a 4 × 4 × 4 super-cell. In order to understand the effect of Al, the calculations were performed on Al_2_Ge_62_Te_64_ model (which is close to the experimental Ge_0.97_Al_0.03_Te composition). The distance between two Al atoms was 12.02 Å. For the irreducible cell, the Brillouin-zone integration was performed using a 25 × 25 × 25 Monkhorst−Pack *k*-mesh. For the super-cell, we used a 3 × 3 × 3 *k*-mesh for the atomic relaxation and a 7 × 7 × 7 *k*-mesh for the electronic density of states (DOS) calculations.

## 3. Results and Discussion

**Figure 1:**
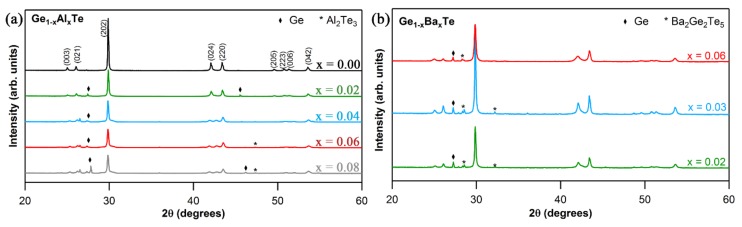
XRD patterns for Ge_1−x_Al_x_Te (**a**) and Ge_1−x_Ba_x_Te (**b**) systems.

The sharp reflections observed from X-ray diffraction (XRD) patterns for Al and Ba doped GeTe (Figure 1a,b, respectively) indicate the crystalline nature of the phases. The main reflections in both cases could be indexed to the rhombohedral (*R*3*m*) GeTe phase (PDF# 00-047-1079). The rhombohedral phase was further confirmed by the presence of double reflections [(0 2 4) and (2 2 0)] in the range of 2θ values between 41° and 44°. A minor proportion of Ge-rich secondary phase was also present, as in agreement with the previous studies [7,8,9]. Based on the evolution of lattice parameters, the solubility limit for Al in GeTe was estimated to be 4 mol%. At higher content of Al (for *x* > 0.04), Al_2_Te_3_ secondary phase started to appear and the GeTe main phase was not rhombohedral anymore (change of symmetry). The solubility limit for Ba in GeTe was found to be minimum (< 2 mol%), as Ba_2_Ge_2_Te_5_ phase existed in all the samples. This is unsurprising given the larger atomic radii of Ba compared to that of Ge.

**Figure 2:**
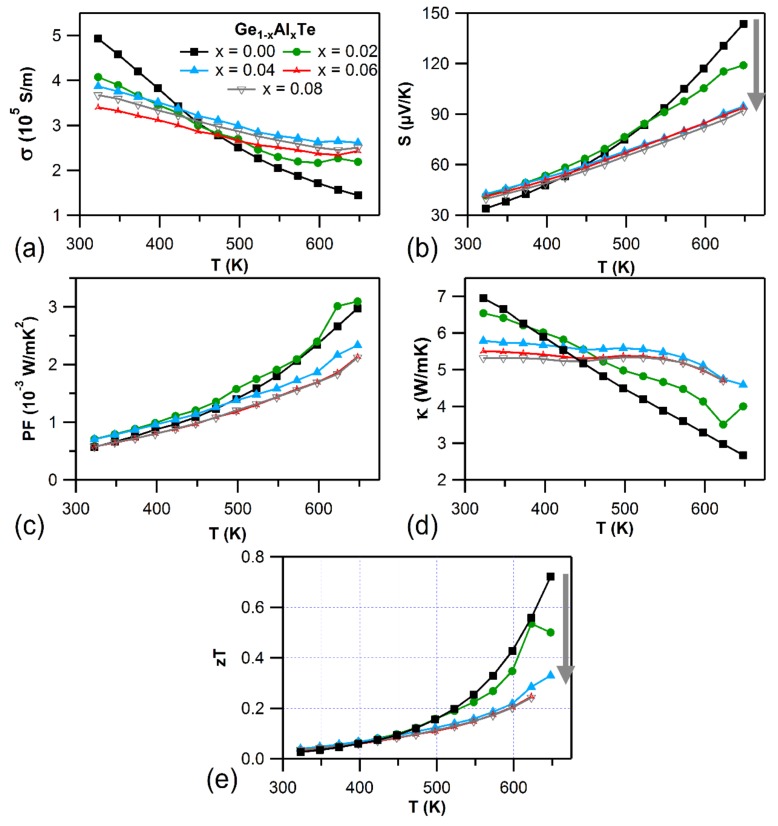
Temperature-dependent (**a**) electrical conductivity (*σ*), (**b**) Seebeck coefficient (*S*), and (**c**) power factor (PF = *S*^2^*σ*), (**d**) total thermal conductivity (*κ*), and (**e**) figure of merit (*zT*) for Ge_1−x_Al_x_Te (*x* = 0.00–0.08) samples.

**Figure 3:**
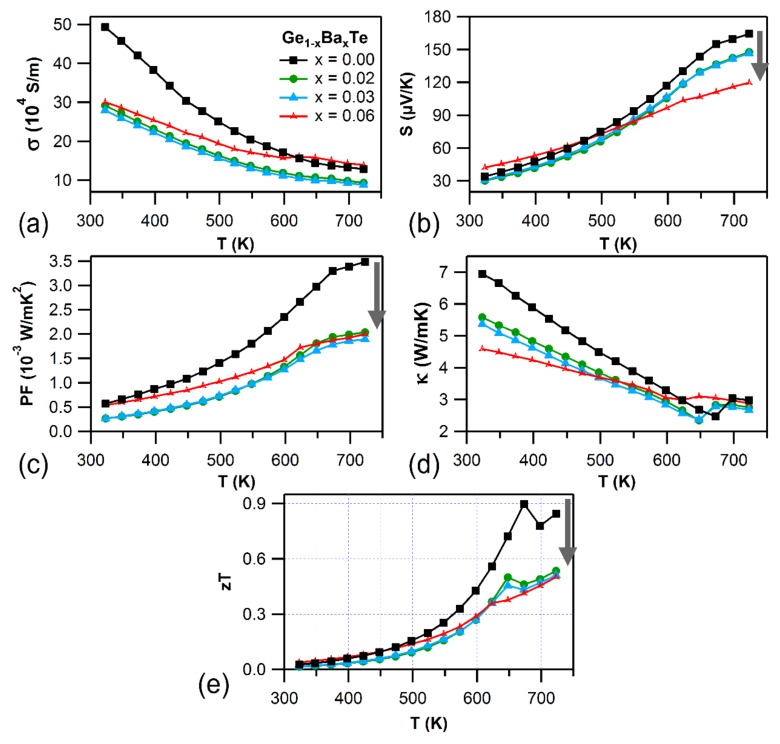
Temperature-dependent (**a**) electrical conductivity (*σ*), (**b**) Seebeck coefficient (*S*), and (**c**) power factor (PF = *S*^2^*σ*), (**d**) total thermal conductivity (*κ*), and (**e**) figure of merit (*zT*) for Ge_1−x_Ba_x_Te (*x* = 0.00–0.06) samples.

Results from Hall measurements tabulating carrier concentration (*n*) and mobility (*µ*) are presented in Table 1. Holes are the major charge carriers (*p*-type), as the Hall voltage was found to be positive (*p*-type) in both Ge_1−x_Al_x_Te and Ge_1−x_Ba_x_Te systems. Doping Al to GeTe provides extra holes to the system, which is reflected in the enhancement in charge carrier density. This is in contrast to the effect observed in In and Ga (isoelectronic with Al) doped GeTe, where In and Ga decreased the hole concentration by filling up Ge vacancies [8,19]. On the other hand, the mobility reduction can be attributed to the alloy scattering mechanism arising from the doping of Al and Ba to GeTe [34]. Due to decreased mobility, the electrical conductivity at room temperature was decreased for both Ge_1−x_Al_x_Te (Figure 2a) and Ge_1−x_Ba_x_Te (Figure 3a) systems with respect to that of GeTe. However, this trend was reversed at higher temperatures (cross over point at ~450 K) for Al-doped GeTe (Figure 2a). Such similar cases were reported for SnTe and PbTe-based materials, and those cross over effects were attributed to the changes in the electronic band structure with increasing temperature [35,36]. The electrical conductivity of all the samples decreased with increasing temperature, which suggests a degenerate semi-conducting behavior. The positive Seebeck coefficient confirmed the *p*-type charge carriers in Al and Ba doped GeTe (Figure 2b and Figure 3b), which was consistent with the Hall measurement results. The thermopowers of Ge_1−x_Al_x_Te and Ge_1−x_Ba_x_Te monotonically increased with temperature. With increasing Al and Ba content, the change in S values at room temperature were not much evident, but they decreased significantly with increasing temperature, when compared to pristine GeTe. Doping of Al and Ba to GeTe decreased the S values, as it drastically inflated the hole carrier concentration. Consequently, the reduction of Seebeck coefficient with Al and Ba content also considerably affected the thermoelectric power factor (Figure 2c and Figure 3c). Finally, with Al and Ba doping, the total thermal conductivity decreased considerably at room temperature (Figure 2d and Figure 3d). However, it remained almost constant with temperature for Al-doped GeTe. The decreased thermopower significantly affected the thermoelectric figure of merit, *zT* (Figure 2e and Figure 3e), which plunged with dopant concentration. 

**Figure 4:**
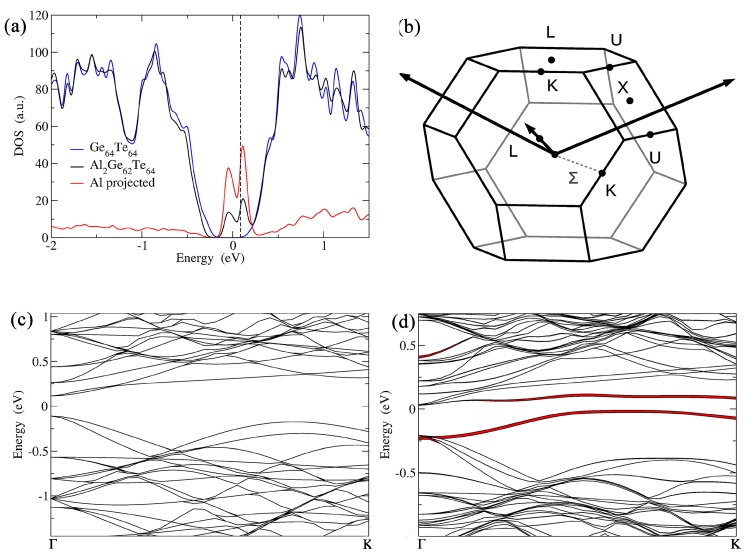
(**a**) Calculated DOS for Al_2_Ge_62_Te_64_ (Ge_0.97_Al_0.03_Te) model, which is compared with that of the pristine cubic phase Ge_64_Te_64_ (c-GeTe). The Fermi level (*E*_F_) of pristine c-GeTe is set arbitrarily at 0 eV. The dashed line represents the shifted *E*_F_ for the doped compositions. Additional Gaussian smearing of 25 meV was applied and the Al projected DOS is magnified for a better readability of the curves. (**b**) Brillouin zone of c-GeTe. Band structures for (**c**) c-Ge_64_Te_64_ using a 4 × 4 × 4 supercell showing band folding in the Γ → K (∑) direction, and (**d**) Al_2_Ge_62_Te_64_ (Ge_0.97_Al_0.03_Te) highlighting Al projections. Line thickness is proportional to the projection of the wave function on the Al (in red) orbitals.

To have a more cogent understanding on the detrimental effects of these dopants in GeTe, DFT calculations were performed. As we were interested in the high temperature domain for thermoelectric application, these DFT calculations were carried out on 4 × 4 × 4 super-cells derived from the cubic structural arrangement of GeTe. The electronic density of states (DOS) computed for the cubic models of pristine and Al-doped GeTe (Al_2_Ge_62_Te_64_ ≈ Ge_0.97_Al_0.03_Te) are shown and compared in Figure 4a. The Al-induced resonant states (distinctly indicated by a sharp hump) are present around the Fermi level (*E*_F_), just like its isoelectronic counterparts In and Ga [8,19]. In such a situation, the Seebeck coefficient is expected to increase, which is not the case with Al (Figure 2b). 

Since the DOS calculations yielded inconclusive evidence, electronic band structures were computed to decipher the role of Al in GeTe. The band structures are plotted in Figure 4 along some high symmetry lines of the cubic Brillouin zone (BZ). The energy difference between light and heavy hole valence bands (ΔE_LΣ_) for undoped cubic Ge_64_Te_64_ was found to be 64 meV, consistent with a recent report [37]. The flat and localized Al bands are located within the principal band bap (Figure 4d). Al-doping in GeTe increased the energy separation between the light hole and heavy hole valence bands to 179 meV (180% increment in ΔE_LΣ_ when compared to pristine c-GeTe), thus disfavoring the band convergence. According to Mott’s relationship, Seebeck coefficient strongly depends on the total DOS effective mass, which in fact is directly proportional to the product of *N_v_*^2/3^ and the average DOS effective mass for each pocket (*N_v_* is the number of degenerate carrier pockets) [38]. For GeTe, *N_v_* is 4 for the L band and it increases to 12 for the ∑ band [21]. Hence, by increasing the energy separation between L and ∑ bands by doping of Al to GeTe, the contributions from the additional carriers (from ∑ valence band) to electrical transport are lost, thus resulting in a significant reduction in the Seebeck coefficient. 

For composition at *x* = 0.02, Al-doped GeTe exhibits a thermopower of ~110 µV/K at 623 K. For the same level of doping, the isoelectronic In-doped GeTe is known to exhibit a much higher thermopower of ~200 µV/K at the same temperature [19]. ΔE_LΣ_ for the In-doped GeTe (for InGe_63_Te_64_ ≈ Ge_0.98_ln_0.02_Te model) is calculated to be 95 meV, which is almost two times lower than the ΔE_LΣ_ for Al-doped GeTe. It must be noted that, even though the ΔE_LΣ_ for In-doped GeTe is marginally higher than that of pristine GeTe, the presence of In-induced resonant states near the *E*_F_ has helped it to exhibit a superior thermopower compared to pristine GeTe. However, for the Al-doped GeTe, the beneficial effect of the presence of Al-induced resonant states near the *E*_F_ is nullified and severely affected by the extremely large energy separation between the light hole and heavy hole valence bands (179 meV). This explains the juxtaposition of high thermoelectric performance of Ge_0.98_In_0.02_Te and low thermoelectric performance of Ge_0.98_Al_0.02_Te (isoelectronic) compounds.

For the case of Ba-doped GeTe, though the DFT results were inconclusive in portraying a clearer picture to explain the reduction in thermopower, it can be attributed to the presence of the secondary phase (Ba_2_Ge_2_Te_5_). More in-depth studies, like experiments to synthesize this Ba_2_Ge_2_Te_5_ phase and measure its transport properties (to estimate the role of contribution of that secondary phase to the overall thermoelectric behavior of the Ge_1−x_Ba_x_Te compound) are required to understand the causes for Ba-doped GeTe to exhibit lower TE performance.

## 4. Conclusions

The crystalline ingots of Ge_1−x_Al_x_Te (*x* = 0–0.08) and Ge_1−x_Ba_x_Te (*x* = 0–0.06) were prepared by the vacuum-sealed tube melting route, followed by Spark Plasma Sintering processing. Al and Ba are found to not be the best choice of dopants for GeTe, as they subside its thermoelectric performance. Al-doping, unlike other isoelectronic group-13 elements (In and Ga), inflates the hole concentration and drastically increases the energy separation between light and heavy hole bands in GeTe, thus resulting in a reduced thermopower. 

## Figures and Tables

**Table 1:** Hall measurement results (at ~300 K) of carrier concentration (*n*) and mobility (*µ*) for Ge_1−x_Al_x_Te (*x* = 0.00–0.08) and Ge_1−x_Ba_x_Te (*x* = 0.00–0.06) samples.

Sample	Carrier Concentration, *n* (cm^−3^)	Mobility, *µ* (cm^2^V^−1^s^−1^)
GeTe	9.08 × 10^20^	57.0
Ge_0.98_Al_0.02_Te	1.75 × 10^21^	21.8
Ge_0.96_Al_0.04_Te	2.88 × 10^21^	10.6
Ge_0.94_Al_0.06_Te	2.17 × 10^21^	12.5
Ge_0.92_Al_0.08_Te	3.01 × 10^21^	8.8
Ge_0.98_Ba_0.02_Te	9.78 × 10^20^	28.2
Ge_0.97_Ba_0.03_Te	9.06 × 10^20^	33.6
Ge_0.94_Ba_0.06_Te	1.62 × 10^21^	16.2
